# Relationship of Anxiety and Depression with Respiratory Symptoms: Comparison between Depressed and Non-Depressed Smokers in Singapore

**DOI:** 10.3390/ijerph16010163

**Published:** 2019-01-08

**Authors:** Cyrus SH Ho, Elysia LY Tan, Roger CM Ho, Marcus YL Chiu

**Affiliations:** 1 Department of Psychological Medicine, National University Health System, Singapore 119228, Singapore; 2 Faculty of Arts and Social Sciences, National University of Singapore, Singapore 117416, Singapore; elysiatanly@gmail.com; 3 Department of Psychological Medicine, Yong Loo Lin School of Medicine, National University of Singapore, Singapore 119228, Singapore; pcmrhcm@nus.edu.sg; 4 Centre of Excellence in Behavioral Medicine, Nguyen Tat Thanh University (NTTU), Ho Chi Minh City 70000, Vietnam; 5 Faculty of Education, Huaibei Normal University, 100 Dongshan Road, Huaibei, Anhui 235000, China; 6 Department of Social & Behavioural Sciences, City University of Hong Kong, Hong Kong, China; mylchiu@cityu.edu.hk

**Keywords:** smoking cessation, depression, anxiety, respiratory symptoms, cardiovascular disease

## Abstract

The rising prevalence of smokers in the community, specifically psychiatric patients, necessitates smoking cessation as an important strategy for reducing the harmful effects of tobacco. This study aims to compare the profiles of depressed and non-depressed smokers and evaluate how psychiatric symptoms influence respiratory symptoms. A cross-sectional survey was administered to 276 non-depressed adult smokers in the community and 69 adult smokers who had been formally diagnosed with depression in the outpatient clinic of a University Hospital in Singapore. Participants were administered questionnaires on smoking attitudes and perceptions, psychiatric symptoms, and respiratory symptoms. Correlations and multiple regression analyses were conducted. The mean age of smokers in the study was 35.32 ± 13.05 years. Smokers in the community and psychiatric samples were largely similar on all of the sociodemographic factors, except that fewer depressed people were employed (χ^2^ = 8.35, *p* < 0.01). Smokers with depression also reported more attempts to quit smoking (χ^2^ = 7.14, *p* < 0.05), higher mean depressive, anxiety, and stress symptom (DASS) scores (t = −10.04, *p* < 0.01), and endorsed more respiratory symptoms than smokers in the community (t = −2.40, *p* < 0.05). The DASS scores, number of cigarettes smoked daily, years of smoking, general perception of smokers getting heart disease, and presence of lung disease were positively and significantly correlated with respiratory symptoms. On multiple regression, only anxiety symptoms (β = 0.26, *p* < 0.05) and the presence of lung disease (β = 0.22, *p* < 0.001) were significantly correlated with respiratory symptoms. Depressed smokers reported greater difficulty in quitting tobacco use, and they perceived more severe respiratory symptoms compared to non-depressed counterparts. Anxiety symptoms were positively associated with the severity of respiratory symptoms. Smoking cessation campaigns need to specifically target psychological symptoms in smokers and focus more psychoeducation on the risk of cardiovascular disease in the middle-aged population.

## 1. Introduction

Tobacco smoking is a global health risk with established high morbidity, mortality, and socioeconomic burden. Ranked as the fourth most common risk factor for disease [1], smoking causes over 20 different diseases ranging from respiratory diseases, such as asthma and chronic obstructive pulmonary disease (COPD), to cardiovascular diseases, stroke, and multiple cancers, including lung cancer. It is also a leading cause of preventable death that amounts to more than seven million deaths occurring worldwide annually [2]. It accounts for 71% of all lung cancer deaths, 36% of respiratory disease deaths, and 10% of all cardiovascular disease-related deaths [3]. It is also associated with a range of psychiatric disorders, of which mood and anxiety disorders are the most prevalent, and individuals with psychiatric disorders are often found to have comorbid nicotine dependence. Smoking is approximately twice as common among people with psychiatric disorders at 40% compared to the general population, and more so in those with severe diseases [4]. Based on population-based prevalence studies, 59% of individuals with major depressive disorder and 60% of those with dysthymia are lifetime smokers [5]. Smokers with comorbid depression tend to be heavier smokers [5] and have a two to three times increased risk of smoking-related morbidity and mortality, such as cardiovascular disease and cancer, compared to non-depressed smokers [6].

Respiratory symptoms such as breathlessness, cough, chest tightness, and wheeze are often a manifestation of respiratory diseases such as asthma and COPD, and occur in the context of smoking. Smoking is related to a greater severity of respiratory symptoms, poorer symptom control, more frequent healthcare utilization, and the decreased effectiveness of commonly used respiratory medications (e.g., inhaled corticosteroids) [7]. Psychological status such as depression and anxiety were also found to be associated with respiratory symptoms [8], although there are limited empirical data delineating the relationship between psychiatric symptoms and respiratory symptoms.

Smoking prevalence in Singapore has been hovering since 2004, with no significant decline over the years despite the implementation of various policies and legislative measures aimed at reducing smoking. These included raising the minimum legal age for the sale, purchase, and possession of tobacco products from 18 to 21 years of age, banning point-of-sale displays, and upscaling smoking cessation programs. According to the National Population Health Survey, the overall prevalence of daily smoking was 12% in 2017, with more males compared to females [9]. Based on the findings of the Singapore Mental Health Survey of 2010, the highest proportion of smokers were in the younger age group, 18 to 34 years of age, and the prevalence of nicotine dependence in the population was 4.5% [1]. Nevertheless, these surveys did not evaluate the impact of depression on smoking and how it influences smoking cessation.

Despite high rates of co-occurrence and the associated morbidity, the treatment of tobacco dependence in depressed individuals remains suboptimal. According to a 2001 study, psychiatric patients received smoking cessation counselling in only 38% of their visits with a primary care physician and 12% of their visits with a psychiatrist [10]. Evidence suggests that clinicians’ lack of active involvement in the promotion of smoking cessation could be related to their perception that depressed smokers were unmotivated to quit, smoking is an attempt at self-medication, and cessation would exacerbate depressive symptoms [11]. However, it is increasing recognized that smokers with psychiatric issues are just as likely to want to quit as the general population, but are more likely to be heavily addicted to smoking, anticipate difficulty quitting smoking, and thus be less likely to succeed in any quit attempt [12].

Therefore, the objectives of this study are to compare the profile of depressed and non-depressed smokers and evaluate how psychiatric symptoms influence respiratory symptoms. We hypothesize that depressed patients face unique challenges that may impede their smoking cessation journey, and that psychiatric symptoms are positively correlated with respiratory symptoms. Thus, it is pertinent to identify the unique factors that can be mitigated and incorporated into future cessation programs.

## 2. Material and Methods

### 2.1. Participants and Procedure

We performed a cross-sectional survey of patients who were on follow-up in the depression clinics of the National University Hospital and people in the community from January to December 2016. Patients were recruited into the study if they were currently smoking, above 21 years old, and formally diagnosed by their psychiatrist to have depression according to the criteria of the fifth edition of the Diagnostic and Statistical Manual (DSM). Those with comorbid psychiatric disorders such as psychotic disorders and personality disorders and with serious medical conditions in immediate need of treatment were excluded. The community sample was age, sex, and ethnicity-matched to the clinical sample in order to limit the confounding effects of these parameters. Individuals were also included if they were current smokers, above 21 years old, and without psychiatric history or serious medical conditions in immediate need of treatment. They were regarded as ‘non-depressed’ if they reported no current or past depressive symptoms, and their depression symptom score on the questionnaire was normal. A total of 69 patients and 207 individuals from the community were recruited into the study in a 1:3 fashion. The interview and self-report questionnaires were administered by trained psychology students from the National University of Singapore. Written consent was obtained from all of the participants, and this study was approved by the National Healthcare Group (NHG) Domain Specific Review Boards (DSRB).

### 2.2. Measures

#### 2.2.1. Sociodemographic Data, Medical History, and Psychiatric History

The sociodemographic data that were collected included ethnicity, education, marital status, employment, income, and living arrangement as surrogates for social support. Details of the participants’ psychiatric and medical history were obtained from self-reported accounts. A detailed list of respiratory symptoms related to smoking was assessed and were considered frequent if they occurred at least three times per week. These symptoms included cough, phlegm, wheeze, blocked or running nose, and the use of medication for asthma/COPD, and these were based on other studies that evaluated smoking-related respiratory symptoms [13]. A composite score of the total number of symptoms that the individual experienced was calculated.

#### 2.2.2. Smoking Attitudes and Perception

The participants’ smoking histories, which included years of smoking, number of cigarettes smoked per day, and past quitting attempts, were included. Two specific questions on smokers’ attitudes on the importance and confidence in quitting smoking were added, and they incorporated a grading scale of 1–100 with ‘1’ denoting the least important/confident and ‘100’ denoting the most important/confident. Three questions on the general perception of smoking on a five-point Likert scale with ‘1’ denoting strongly disagree and ‘5’ denoting strongly agree were included: ‘Smokers live shorter lives than non-smokers’, ‘Smoking increases your chance of getting lung cancer’, and ‘Smokers are more likely to get heart disease’.

#### 2.2.3. Depression, Anxiety, and Stress Scale (DASS-21)

The DASS-21 is a 21-item self-administered questionnaire that contains three subscales for depression, anxiety, and stress, each with seven items. The depression subscale evaluated dysphoria, anhedonia, and hopelessness. The anxiety subscale measured autonomic arousal, situational anxiety, and subjective experience of anxiety. The stress subscale was sensitive to chronic non-specific arousal and evaluated domains of tension, nervous arousal, agitation, irritability, and impatience. Scores for depression, anxiety, and stress were calculated by summing the scores for the relevant items. It has been demonstrated to be a good psychometric screening tool with good validity and reliability [14].

#### 2.2.4. Fagerstrom Test for Nicotine Dependence (FTND)

This is a six-item test that evaluates the physical dependence on tobacco smoking. Each question was given a score of zero, one, two, or three depending on the response options, and they were summed to give a total score that ranged from zero to ten. The higher the overall score, the more likely the individual will have prominent withdrawal symptoms if they were to quit smoking. Having been applied in various settings, it has shown good validity and reliability [15].

### 2.3. Statistical Analyses

All of the data were analyzed using the IBM Statistical Package for the Social Sciences (SPSS) (version 23, Armonk, New York). Means, standard deviations, frequencies, and percentages were calculated for demographic statistics. Chi-square and *t*-tests were used to compare differences between the patient and community groups. Correlation analysis was conducted to examine the relationship between respiratory symptoms and various potential predictors in smoking behaviors and attitudes and psychiatric symptoms. Multiple regression was further conducted to evaluate whether the group, number of cigarettes smoked daily, years of smoking, general perception of smokers getting heart disease, presence of lung disease, and DASS scores were all necessary to predict respiratory symptoms. As age, gender, and ethnicity were matched between groups, these variables were not added into the model as covariates. Education, marital status, and employment were initially added into the model as covariates; however, they did not significantly change the model, and were removed from the final analysis. For all of the statistical analyses, the significance threshold was set at *p* < 0.05.

## 3. Results

Table 1 summarizes the descriptive comparison between the two groups (clinical and community sample) on measures of attitudes, behavior of smoking, depression, anxiety, and stress symptoms, as well as sociodemographic factors. The mean age of smokers in the study was 35.32 ± 13.05 years, with the clinical and community samples having mean ages of 36.07 ± 13.16 years and 35.07 ± 13.03 years respectively. There was a higher proportion of females (60.9%) in the study. The groups did not differ in all of the sociodemographic factors except for employment: more people were significantly employed in the community sample (χ^2^ = 8.35, *p* < 0.01). The depressed group that was recruited from psychiatric clinics had severe depressive symptoms (*M* = 22.17, SD = 11.36), severe anxiety symptoms (*M* = 17.81, SD = 10.31), and moderate stress symptoms (*M* = 23.78, SD = 10.69). Conversely, the non-depressed group that had been recruited from the community reported normal levels of depressive and stress symptoms, but had mild anxiety symptoms (*M* = 9.32, SD = 8.65).

Table 2 highlights the statistical comparison between the two groups on respiratory and smoking-related variables. There were no statistical differences in their years of smoking, presence of lung disease, and general perception of smoking. However, they were significantly different in the number of times that they had previously tried to quit smoking, DASS scores, and composite scores of the respiratory symptoms. The clinical sample reported having more attempts to quit (χ^2^ = 7.14, *p* < 0.05), higher DASS scores (t = −10.04, *p* < 0.01), and endorsed more respiratory symptoms than the community sample (t = −2.40, *p* < 0.05).

Table 3 summarizes the correlations between DASS and respiratory symptoms with smoking behaviors, attitudes, and social demographics. The three components of the DASS scores were positively and strongly correlated with one another (*p* < 0.01). All three components (Stress: r = 0.22, *p* < 0.01; Anxiety: r = 0.29, *p* < 0.01; Depression: r = 0.24, *p* < 0.01) and total DASS scores (r = 0.262, *p* < 0.01) were significantly and positively correlated with respiratory symptoms, indicating that those with higher scores on these variables tended to endorse more respiratory symptoms. Respiratory symptoms were strongly correlated with the presence of lung disease (r = 0.24, *p* < 0.001), and there was no significant association between DASS scores and lung disease. Having a general perception of smokers getting heart disease was significantly and positively correlated with total DASS scores (r = 0.135, *p* < 0.05), and this relationship was also observed with the anxiety component of DASS (r = 0.12, *p* < 0.05).

Table 4 shows the multiple regression of DASS symptoms, number of cigarettes smoked daily, years of smoking, group, general perception of getting heart disease, and presence of lung disease onto respiratory symptoms. In model one of the analysis, group was dummy coded as zero or one (community or clinical) and entered into the regression equation. This model showed that the group explained 1.6% of the variance and was significantly and positively related to respiratory symptoms (*R*^2^ = 0.016, *F*(1, 274) = 4.49, *p* < 0.05). Model 2 showed that the adding number of cigarettes smoked daily, years of smoking, and general perception of smokers getting heart disease into the model explained 1.8% of the variance in respiratory symptoms (*R*^2^ = 0.018, *F*(3, 271) = 0.14, *p* < 0.001). Model 3 showed that adding the presence of lung disease into the model explained 7% of the variance in respiratory symptoms (*R*^2^ = 0.070, *F*(1, 270) = 15.24, *p* < 0.001), and that the group was no longer significantly correlated with respiratory symptoms. Model 4 showed that adding DASS scores into the model explained 11.2% of the variance in respiratory symptoms (*R*^2^ = 0.112, *F*(1, 269) = 12.74, *p* < 0.001), and the presence of lung disease was still significantly correlated with respiratory symptoms. In addition, DASS scores were also significantly correlated with respiratory symptoms.

In Table 5, the results after the DASS subscales were separately put into model four of the analysis are shown. Model four, which added the three DASS subscales (anxiety, depression, and stress), explained 12% of the variance in respiratory symptoms (*R*^2^ = 0.10, *F*(3, 267) = 5.48, *p* < 0.001). The presence of lung disease (β = 0.22, *p* < 0.001) and anxiety (β = 0.26, *p* < 0.05) were significantly correlated with respiratory symptoms, but not depression and stress.

## 4. Discussion

### 4.1. Depressive and Anxiety Symptoms between the Clinical and Community Groups

This is one of the few studies that attempted to compare the smoking profile between depressed patients and community individuals and explore the relationship between psychiatric symptoms and respiratory symptoms. In this study, we found that the depressed group recruited from psychiatric clinics had severe anxiety symptoms, and the non-depressed group recruited from the community also had mild anxiety symptoms. This highlights that anxiety symptoms are often prominently comorbid in depressive disorders, and that anxiety symptoms, albeit mild, do occur within the community. This is especially so in smokers, as tobacco smoking has been associated with anxiety and depressive symptoms of varying degrees [16]. Our study revealed that more depressed patients were unemployed than those in the community, which was consistent with studies demonstrating the impact of depression in impairing work function, causing disability and increasing economic burden [17].

### 4.2. Factors Contributing to Smoking in Depressed Smokers

Depressed individuals in our study reported more difficulty quitting smoking and had more failed attempts, and this has been similarly reflected in other studies that highlighted the positive association of depressive symptoms with smoking severity and poorer smoking cessation outcomes [18]. People with mood instabilities were more likely to drop out from substance abuse treatment and suffer quicker relapse to smoking after a quit attempt [18]. The possible reasons contributing to these phenomena are multifold. Nicotine can temporarily improve mood and cognitive function, enhance vigilance, and reduce stress, possibly via the release of dopamine in the prefrontal cortex, thereby regulating dysfunction of the mesolimbic and mesocortical dopamine systems that occurs in depression [19]. With the positive psychological and cognitive effects resulting from the neurochemical changes induced by nicotine, smoking may epitomize a form of self-medication [20]. These effects may also positively reinforce the need to continue smoking. Studies have demonstrated smoking’s ability to improve psychomotor and physiological functioning in depressed patients [19], although the benefits were temporary and relapse occurred shortly after smoking cessation, and depressive symptoms worsened with nicotine withdrawal [21]. The inhibition of monoamine oxidase by constituents in tobacco can also possibly enhance the addiction of tobacco smoking [22]. Smoking may also be a marker of more severe illness processes, as evidenced by smokers having an earlier onset of illness, increased frequency of hospitalizations, and receiving higher doses of neuroleptic medication [23]. Shared genetic factors predisposing people to both smoking and depression have also been uncovered in twin studies [24], suggesting genetic predisposition in the initiation and maintenance of smoking in depressed patients.

### 4.3. Relationship of Anxiety Symptoms to Perceptions of Cardiovascular Disease

Higher DASS scores and, in particular, anxiety symptom scores were positively associated with having the general perception that smokers are more likely to get heart disease. However, this was not observed with the other perceptions, including smokers living shorter lives and increasing chances of lung cancer, suggesting cardiovascular disease to be a more pertinent concern among smokers compared to lung cancer and life expectancy. This is not unexpected, considering that cardiovascular disease is the leading cause of smoking-related deaths worldwide, accounting for nearly half of all smoking-related deaths in industrialized nations [25]. Interestingly, the presence of lung disease was not significantly associated with anxiety or other psychiatric symptoms. Based on epidemiological studies, lung cancer only accounted for less than a quarter of all smoking-related deaths [26], despite smoking being the largest cause of lung cancer deaths worldwide [3]. For people younger than 45 years old, cardiovascular disease was the predominant cause of increased mortality attributable to cigarette smoking, while mortality from lung cancer increased sharply only after 50 years old [27]. Furthermore, cardiovascular disease is more common than lung cancer, and symptoms such as heart attack may be more traumatic and anxiety-provoking, thereby contributing to the perceptions observed in smokers and possibly inducing increased anxiety among smokers.

### 4.4. Relationship between Anxiety Symptoms and Respiratory Symptoms

Significantly higher DASS scores and more respiratory symptoms were reported in the depressed patients than in the non-depressed community sample in this study. All three components of the DASS were also positively associated with respiratory symptoms with the correlation analysis. However, after multiple regression, only anxiety symptoms and the presence of lung disease remained significantly and positively correlated with respiratory symptoms. This suggested that anxiety symptoms were the main driving force associated with respiratory symptoms other than lung disease. Multiple studies have investigated the association of anxiety and depression with respiratory disorders such as asthma [28] and COPD [29]. They have also highlighted the impact of both anxiety and depression on respiratory symptoms, with one study demonstrating an increased probability of manifesting respiratory symptoms with increased Hospital Anxiety Depression Scale (HADS) scores [30], although that study summed the depression and anxiety scale to obtain a combined measure of psychological status. However, few studies have explicitly explored the relationship of depression and anxiety as separate entities with respiratory symptoms, which may be important in targeting smoking cessation strategies. There are two conceivable ways to interpret our findings. The first is that anxiety symptoms cause or contribute to respiratory symptoms, for instance by intensifying the subjective sensation of breathlessness, as evidenced by a study that correlated resistive loads and peak inspiratory mouth pressure to be appreciably reduced in anxious patients compared to normal subjects [31]. Hyperventilation and the panic attacks that form part of anxiety can contribute to respiratory difficulties such as shortness of breath and dyspnoea. Anxiety sensitivity, a transdiagnostic emotional vulnerability that has been described as the fear of anxiety-relevant sensations, has been identified as a potential individual factor that is implicated in the induction, progression, maintenance, cessation, avoidance, and reversion of smoking [32]. It encompasses the fear of undergoing interoceptive sensations of arousal or other anxiety symptoms because the sufferer believes that these experiences indicate or will result in cardiac arrest or other imminent outcomes [32]. This results in a vicious cycle of anticipation, fear, and avoidance, and can trigger more anxiety symptoms and physiological arousal symptoms. This point may also help explain the earlier observation that was mentioned of increased anxiety associated with an increased general perception of smokers having heart disease. Higher anxiety sensitivity has been associated with increased lower respiratory symptoms and a greater risk of smoking relapse [33]. Smokers with respiratory symptoms who had heightened anxiety sensitivity were also more emotionally susceptible to somatic sensations and life stressors, which was possibly due to a dysfunctional cognitive–affective reaction to threatening situations [34], thereby contributing to negatively reinforced behavior such as continued smoking. The second interpretation is that respiratory symptoms cause or contribute to anxiety symptoms, and many studies have also supported this direction of the causality of the relationship. For instance, Goodwin et al. found that asthma was associated with a significantly increased risk of having anxiety disorders [35]. However, the common cofactor between anxiety and respiratory symptoms remains smoking, which directs these two sets of symptoms in a vicious cycle. Furthermore, the association between respiratory and anxiety symptoms is likely to be bidirectional and multiplicative, and may be a product of shared risk factors (genetics, smoking, environment, etc). Nevertheless, as this was a cross-sectional study, we were unable to elucidate the causal associations and directionality of the relationship.

### 4.5. Influence on Targeted Psychiatric Treatment and Smoking Cessation Strategies

In this study, concerns about cardiovascular disease were highlighted as a prominent concern in smokers, as reflected by more participants concurring that smoking can lead to heart diseases, and were found to be correlated with anxiety symptoms. Therefore, smoking cessation programs should focus their psychoeducation on the risk of cardiovascular disease, especially for smokers in the middle-age group, and empower them to better manage their cardiovascular risk factors to reduce anxiety levels. It should be reiterated that smoking more than doubles the risk of all types of cardiovascular disease in a dose-dependent manner, and smoking cessation can be associated with a health benefit within six months [36]. Screening for psychiatric symptoms, particularly anxiety, should form part of comprehensive smoking cessation programs, even in those without prior psychiatric diagnoses, and timely interventions should be administered if symptoms are present (e.g., referral to psychiatric services, counseling), as anxiety is found to significantly correlate with respiratory symptoms regardless of whether depression is present or not. For smokers with psychiatric disorders, clinicians should be more direct in asking patients about their interest in quitting smoking instead of merely attributing their smoking to self-medication coping mechanisms. Instead, more support within hospital and community services must be provided to them, as they may encounter more difficulties with quitting and risk the relapse of psychiatric symptoms during the smoking cessation process.

### 4.6. Limitations and Future Directions

While this was one of the few studies exploring the relationship of psychiatric symptoms with respiratory symptoms in depressed versus non-depressed smokers, there were several limitations that restrict the generalizability of the findings. This was a cross-sectional study, and was thus unable to infer causal relationships. Our clinical sample was only obtained from one general hospital and only included patients with a depression diagnosis. It would also be worthwhile to include smokers with anxiety disorders to delineate the effects of anxiety and depressive symptoms on respiratory symptoms. Our sample was predominantly male, and consisted of middle-aged adults instead of across the age spectrum, thereby preventing generalizability to diverse groups of smokers. We were also unable to have a direct 1:1 comparison of participants in the clinical and community groups. All of the symptoms (psychological or physical) were self-reported, which may be prone to response bias, and there were no clinical measurements, such as spirometry, to support their physical symptoms. Furthermore, other relevant moderators or mediators of psychiatric and respiratory symptoms, such as personality characteristics, medications, and various medical comorbidities, were not documented. There was also no sample size calculation to determine the power in identifying differences between groups. Despite these limitations, this study provides further understanding of the relationship of psychiatric symptoms with respiratory symptoms, which may help direct the focus of smoking cessation programs and provide evidence-based timely intervention. Future studies should also include smokers across the age spectrum and identify the unique factors limiting their smoking cessation.

## 5. Conclusions

Depressed smokers reported greater difficulty in quitting tobacco use, and they perceived more severe respiratory symptoms compared to non-depressed counterparts. Anxiety symptoms were particularly positively associated with the severity of respiratory symptoms. Effective tailored smoking cessation campaigns need to specifically manage psychological symptoms in smokers and focus more psychoeducation on the risk of cardiovascular disease in the middle-aged population.

## Figures and Tables

**Table 1:** Profiles of Respondents (*N* = 276) (Cases Matched 1:3).

Variable	Total *N* = 276 (Valid %)	Depression *n* = 69 (Valid %)	Community *n* = 207 (Valid %)
Age of Respondents (Mean, SD)	35.32 (13.05)	36.07 (13.16)	35.07 (13.03)
Sex			
Male	168 (60.9)	42 (60.9)	126 (60.9)
Female	108 (39.1)	27 (39.1)	81 (39.1)
Ethnicity			
Chinese	212 (76.8)	53 (76.8)	159 (76.8)
Malay	32 (11.6)	8 (11.6)	24 (11.6)
Indian	16 (5.8)	4 (5.8)	12 (5.8)
Others	16 (5.8)	4 (5.8)	12 (5.8)
Education			
Primary	28 (10.1)	11 (15.9)	17 (8.2)
Secondary and Technical Education	110 (39.9)	30 (43.5)	80 (38.6)
Diploma and University	131 (47.5)	28 (40.6)	103 (49.8)
Postgraduate	7 (2.5)	0 (0.0)	7 (3.4)
Marital Status			
Single	169 (61.2)	42 (60.9)	127 (61.4)
Married	90 (32.6)	18 (26.1)	72 (34.8)
Separated/Divorced	15 (5.4)	9 (13.0)	6 (2.9)
Widowed	2 (.7)	0 (0.0)	2 (1.0)
Living Arrangement			
Lives Alone	27 (9.8)	9 (13.0)	18 (8.7)
Lives with Family	212 (76.8)	46 (66.7)	166 (80.2)
Lives with Spouse	9 (3.4)	0 (0.0)	9 (4.3)
Lives with Children	3 (1.1)	1 (1.4)	2 (1.0)
Lives with Spouse and Children	25 (9.1)	13 (8.8)	12 (5.8)
Currently Employed	194 (70.3)	39 (56.5)	155 (74.9)
Monthly Personal Income			
</=$1000	103 (37.3)	39 (56.5)	64 (30.9)
$1000–$2000	64 (23.2)	11 (15.9)	53 (25.6)
$2000–5000	92 (33.3)	17 (24.6)	75 (36.2)
>$5000	17 (6.2)	2 (2.9)	15 (7.2)
Monthly Household Income			
</=$1000	32 (11.6)	12 (17.4)	20 (9.7)
$1000–$2000	51 (18.5)	13 (18.8)	38 (18.4)
$2000–5000	97 (35.1)	20 (29.0)	77 (37.2)
>$5000	95 (34.5)	23 (33.3)	72 (34.8)
Years Smoking (Mean, SD)	15.29 (13.35)	16.69 (14.17)	14.82 (13.07)
Number of Cigarettes Smoked (Mean, SD)	1.45 (0.621)	1.48 (0.66)	1.43 (0.61)
Tried to Quit	162 (58.7)	48 (69.5)	114 (55.1)
Fagerstrom Nicotine Dependence Test (FNDT; Mean, SD)	3.28 (2.96)	3.50 (2.68)	1.90 (1.72)
Depressive, Anxiety, and Stress Symptoms (DASS) (Mean, SD)	36.30 (30.30)	63.77 (28.68)	27.55 (24.99)
Respiratory Symptoms Composite (Mean, SD)	1.69 (1.88)	2.16 (1.96)	1.53 (1.84)
Confidence in Quitting (Mean, SD)	47.49 (31.86)	47.43 (34.74)	47.51 (30.92)
Smokers Live Shorter Lives than Non-Smokers (Mean, SD)	3.12 (1.41)	3.07 (1.44)	3.14 (1.40)
Smoking Increases Chance of Getting Lung Cancer (Mean, SD)	3.82 (1.24)	3.86 (1.32)	3.80 (1.22)
Smokers are More Likely to Get Heart Disease (Mean, SD)	3.59 (1.24)	3.61 (1.31)	3.58 (1.22)
Presence of Lung Disease	41 (14.9)	14 (20.3)	27 (13.0)

**Table 2:** Comparison between Clinical and Community Sample on Respiratory and Smoking-Related Variables.

Variable	*t*-Test	Chi-Square
Years Smoking	−1.01	
Number of Cigarettes Smoked		0.49
Tried to Quit		7.14 *
Fagerstrom’s Nicotine Dependence Test (FNDT)	−0.74	
DASS	−10.04 **	
Respiratory Symptoms Composite	−2.40 *	
Confidence in Quitting	0.016	
Smokers Live Shorter Lives than Non-Smokers	0.35	
Smoking Increases Chance of Getting Lung Cancer	0.31	
Smokers are More Likely to Get Heart Disease	−0.17	
Presence of Lung Disease		2.15

* *p* < 0.05, ** *p* < 0.01.

**Table 3:** Correlation between DASS (DS) and Respiratory Symptoms with Smoking Behaviors, Attitudes, and Social Demographics.

Variable	DS_ Stress	DS_ Anxiety	DS_ Depression	DS_ Total	Respiratory Symptoms	Years Smoke	Number Cigarettes Smoke Daily	FTND	Smokers Live Shorter Lives	Smoking Increases Chance of Lung Cancer	Smokers Are More Likely to Get Heart Disease	Importance of Quitting Smoking	Confidence to Quit Smoking	Age	Sex	Presence of Lung Disease
DS_Total	0.94 **	0.91 **	0.95 **	1	0.262 **	−0.101	0.070	0.017	0.090	0.075	0.135 *	−0.009	−0.005	−0.128 *	0.051	0.10
Respiratory Symptoms	0.22 **	0.29 **	0.24 **	0.262 **	1	−0.081	0.050	0.104	0.036	0.011	0.014	0.073	−0.038	−0.115	0.068	0.24 ***
DS_Stress	1	0.77 **	0.85 **	0.94 **	0.22 **	−0.065	−0.058	0.019	0.10	0.11	0.15	−0.006	0.017	−0.082	0.068	0.086
DS_Anxiety	0.77 **	1	0.79 **	0.91 **	0.29 **	−0.17 **	−0.10	−0.003	0.071	0.050	0.12 *	0.038	0.012	−0.20 **	0.067	0.11
DS_Depression	0.85 **	0.79 **	1	0.95 **	0.24 **	−0.061	−0.039	0.030	0.076	0.050	0.11	−0.049	−0.040	−0.092	0.011	0.091

* *p* < 0.05, ** *p* < 0.01, *** *p* < 0.001.

**Table 4:** Regressing DASS (DS) Symptoms, Number of Cigarettes Smoked Daily, Years of Smoking, Group, General Perception of Getting Heart Disease, and Presence of Lung Disease onto Respiratory Symptoms.

Variable	Model 1			Model 2			Model 3			Model 4		
	*B*	*SE B*	*β*	*B*	*SE B*	*β*	*B*	*SE B*	*β*	*B*	*SE B*	*β*
Group	0.89	0.42	0.13 *	0.90	0.42	0.13 *	0.74	0.41	0.11	−0.15	0.48	−0.021
No. Cig Daily				0.009	0.32	0.002	0.049	0.31	0.010	0.092	0.31	0.019
Years Smoking				−0.008	0.015	−0.035	0.0001	0.015	−0.002	0.004	0.014	0.018
Get Heart Disease				0.031	0.15	0.012	0.028	0.15	0.012	−0.038	0.15	−0.016
Lung Disease							10.99	0.51	0.23 ***	10.91	0.50	0.22 ***
DS_Total										0.025	0.007	0.25 ***
*R* ^2^	0.016			0.018			0.070			0.112		
*F* for change in *R*^2^	4.49 *			0.14			15.24 ***			12.74 ***		

* *p* < 0.05, ** *p* < 0.01, *** *p* < 0.001.

**Table 5:** Regressing Depression, Anxiety, and Stress Symptoms, Group, and General Perception of Getting Heart Disease onto Respiratory Symptoms.

Variable	Model 1			Model 2			Model 3			Model 4		
	*B*	*SE B*	*β*	*B*	*SE B*	*β*	*B*	*SE B*	*β*	*B*	*SE B*	*β*
Group	0.89	0.42	0.13 *	0.904	0.422	0.129 *	0.74	0.41	0.11	0.081	0.490	0.012
No. of Cig Daily				0.003	0.317	0.001	0.049	0.31	0.010	0.104	0.304	0.021
Years of Smoking				−0.008	0.015	−0.037	0.0001	0.015	−0.002	0.008	0.014	0.035
Get Heart Disease				0.031	0.15	0.012	0.028	0.15	0.012	−0.021	0.145	−0.009
Lung Disease							10.99	0.51	0.23 ***	10.885	0.497	0.221 ***
DS_Stress										−0.009	0.032	−0.033
DS_Anxiety				0.018			0.070			0.082	0.031	0.264 *
DS_Depression				0.14			15.24 ***			0.006	0.033	0.022
*R* ^2^	0.016			0.017			0.070			0.12		
*F* for change in *R*^2^	4.49 *			0.18			15.24 ***			5.48 **		

* *p* < 0.05, ** *p* < 0.01, *** *p* < 0.001.
